# Cost, research, and education: Providers’ perspectives on making neurofeedback more accessible

**DOI:** 10.21203/rs.3.rs-6414373/v1

**Published:** 2025-05-07

**Authors:** Whitney K. Norris, M. Kathryn Allison, Linda Larson-Prior, Jocelyn C. Anderson, Martha Rojo

**Affiliations:** University of Arkansas for Medical Sciences; University of Arkansas for Medical Sciences; University of Arkansas for Medical Sciences; University of Arkansas for Medical Sciences; University of Arkansas for Medical Sciences

**Keywords:** neurofeedback, EEG biofeedback, implementation science, Consolidated Framework of Implementation Research, accessibility

## Abstract

**Background:**

Despite a wide variety of research and clinical utilization over the past 50 years, neurofeedback has failed to gain recognition comparable to other well-researched mental health interventions. The goal of this study was to explore neurofeedback practitioners’ perspectives on what would help make the intervention more accessible to mental health providers and to the public.

**Methods:**

As part of a larger implementation study using mixed methods to identify determinants of neurofeedback implementation, this qualitative study utilized a portion of semi-structured qualitative interviews with neurofeedback practitioners practicing in an outpatient setting (n = 17) to specifically explore participants’ ideas around increasing accessibility of the intervention.

**Results:**

The sample was mostly White (n = 15, 88%) and female (n = 13, 76%) with an average age of 53 years (range: 34–73 years). They averaged 17.8 years in practice (range: 3–36 years) and 8 years practicing neurofeedback (range: 1–20 years). Three major themes to help increase accessibility were identified: (a) financial support, including insurance coverage; (b) better provider education, including introduction into graduate school curriculum; and (c) more research/research funding.

**Conclusion:**

Study participants described a variety of specific strategies to make neurofeedback more readily available in routine outpatient mental healthcare. These findings reveal avenues to increase the uptake of neurofeedback in mental healthcare and future directions for implementation science research in the neurofeedback field.

## Introduction

Electroencephalogram (EEG) neurofeedback is a type of biofeedback that uses the electrical activity at the surface of the brain (EEG signals) to provide feedback thought to reinforce specific brain functions through operant conditioning. Through specialized equipment and software, this feedback guides the brain toward different functional goals determined my client and clinician. For example, it has been hypothesized that children with attention-deficit hyperactivity disorder (ADHD) have excess theta waves in their EEG (Van Doren et al., 2019). Thus, the most common neurofeedback protocols used in treating ADHD to date have focused on lowering the prevalence of theta waves in an effort to decrease ADHD symptoms thought to be related to theta activity.

Neurofeedback originally gained attention in the scientific community in the 1950’s and 1960’s when it was being used to successfully treat seizure activity (Sterman et al., 1969, 1974; Sterman & Friar, 1972). Eventually, the intervention gained traction in the mental health community in the treatment of ADHD most notably in the 1990’s (Bluschke et al., 2016; Kuznetsova et al., 2022; Louthrenoo et al., 2022; Moreno-García et al., 2022; Rahmani et al., 2022; Van Doren et al., 2019). Over the past 30 years, the exploration of neurofeedback has expanded to include a wide variety of disorders and symptom presentations within the mental health field (Hammond & Novian, 2017). This includes utilization of neurofeedback to treat depression (Dobbins et al., 2023; Patil et al., 2023; Trambaiolli et al., 2021), anxiety (Hardt & Kamiya, 1978; Micoulaud-Franchi et al., 2021; Tolin et al., 2020), obsessive-compulsive disorder (Zafarmand et al., 2022), personality disorders (Babaskina et al., 2023; Dalkner et al., 2017; Peniston & Kulkosky, 1990), substance abuse (Dave & Tripathi, 2023; Fielenbach et al., 2018; Scott et al., 2005; Sokhadze et al., 2008), and post-traumatic stress disorder (Askovic et al., 2023; Choi et al., 2023; Currie et al., 2014; Gapen et al., 2016; Leem et al., 2021; Panisch & Hai, 2020; Peniston & Kulkosky, 1991; Rogel et al., 2020; Van Der Kolk et al., 2016).

Despite the breadth and variety of literature addressing the use of neurofeedback as a successful intervention, neurofeedback continues to be largely unknown to individuals within the mental health field and the public (Larson et al., 2010). Thus far, there has been limited research bridging the gap between the implementation of neurofeedback in the research setting and the mental health clinic setting. A recent “call to action,” [blinded for peer review] encouraged implementation research on neurofeedback to better understand the barriers and facilitators to implementing neurofeedback in real world settings. The field of Implementation Science offers tools and frameworks to explore such determinants of implementation and strategies to support uptake and sustainability. For example, the Consolidated Framework for Implementation Research (CFIR) is a framework used to guide systematic assessment of potential facilitators and barriers to implementation of interventions like neurofeedback (Damschroder et al., 2009). Notably, there has been no exploration of neurofeedback from an implementation science perspective, despite the potential of implementation science to help increase the uptake of neurofeedback. This study sought to fill that gap by posing the following research question to practicing neurofeedback providers: What would help increase the accessibility of neurofeedback to the public?

## Methods

### Participants

This study was part of a larger mixed methods study that assessed a variety of factors associated with the implementation of neurofeedback in outpatient mental health treatment. The qualitative aim of the larger study involved semi-structured, one-to-one interviews with neurofeedback practitioners. Using a semi-structured interview guide informed by the CFIR, interviews explored practitioners’ process of implementing neurofeedback into practice and their perceptions of implementation facilitators and barriers in the outpatient mental health setting. This paper focuses on one question embedded in the interviews about neurofeedback providers’ perspectives on how to make neurofeedback more accessible to the public. The sample consisted of 17 practitioners implementing neurofeedback in their outpatient mental health care practices who responded to an interview question assessing ways to make neurofeedback more accessible to the public.

### Procedures

This study was approved by the [University blinded for peer review] Institutional Review Board (IRB; Protocol #XXXXXX). Practitioners were first recruited via email through listservs of licensed psychotherapists (Tennessee, Ohio, Maine, Michigan, North Carolina, Rhode Island, and Wyoming). After completing the online survey for the quantitative arm of the parent study, survey participants were asked if they were interested in participating in an interview about their experience with neurofeedback. Second, interview participants were recruited through two recruitment emails sent to the EEG Education and Research (EEGer) company mailing list. EEGer is a training and neurofeedback software company based in the U.S. (www.eeger.com). When potential participants expressed interest in participating in the qualitative interviews, they were sent a link to a brief screener and demographics survey automatically via REDcap. This screener included questions that ensured the participant met the inclusion criteria bullet listed below:

- Have active license to practice psychotherapy
- Currently practicing neurofeedback in an outpatient setting
- Have been implementing neurofeedback in their practices for at least 6 months

Once potential interview participants completed the screener and demographics survey, they were contacted by the PI to schedule their interview. All interviews in the qualitative aim of the parent study were conducted via Zoom, audio recorded and lasted 20-to-40 minutes. Verbal consent to participate was obtained at the start of each interview. All interviews were transcribed verbatim using a transcription service (Otter, https://otter.ai/) and checked for accuracy by the PI (XX). For their participation, interview participants were emailed a discount code for a webinar series geared toward experienced neurofeedback practitioners provided by EEGer (approximately $75 value).

A semi-structured interview guide, informed by CFIR constructs, was developed to assess participants’ perceptions of implementation determinants in the outpatient mental health setting. Questions about accessibility of neurofeedback services and strategies to improve accessibility were not included in the initial interview guide. However, over the first five interviews, participants commonly brought up this issue in describing their experiences of the barriers to implementing neurofeedback, which subsequently led to rich data about participants’ ideas around accessibility and the future of the implementation of neurofeedback. In line with the parent study’s grounded theory techniques, the question, “What would make neurofeedback more accessible to the public?” was then added to the interview guide. The participants who were not initially asked the accessibility question were contacted to request a second interview including only this question. Three of the five participants contacted for this purpose responded resulting in a final sample of 17 for this portion of the analysis. These additional interviews lasted between five to 15 minutes. Any data outside of the accessibility question portion of the interview that applied to the topic of accessibility, for example when discussing the broader barriers to implementation, was also included in the analysis.

### Data Analysis

All interviews were audio recorded and transcribed verbatim using a transcription service (Otter). All transcripts were checked for accuracy by the PI. Then, the research team analyzed all text data using MAXQDA qualitative software. Data used for this analysis consisted of responses to the following question, which were a part of the larger participant interview: “What would make neurofeedback more accessible to the public?”

Thematic analysis was conducted by the first (XX) and second author (XX) to code responses to the added item discussed in this paper about accessibility. Open coding (Sundler et al., 2019) was used to create an inductive thematic codebook based on participants’ responses to the open-ended question about who to make neurofeedback more accessible to the public. After development of the initial codebook, the primary qualitative research expert on the research team (the last author) provided feedback and the research team made alterations to the codebook collaboratively during group discussion. To ensure trustworthiness, multiple coders (XX and XX) participated throughout the entire coding process as codes were refined and reviewed for accuracy and consistency.

## Results

Our sample had an average age of 53 years (range: 34–73 years) and was mostly White (n = 15, 88.24%). Most interview participants identified as female (n = 13, 76.47%). Participants averaged approximately 8 years practicing as a neurofeedback provider (range: 1–20 years) and about 17.6 years in practice (range: 3–36 years). Most worked in solo private practices (n = 11, 64.71%), with two participants (11.76%) working in group private practice, two (11.76%) in community mental health agencies and, two (11.76%) in other outpatient settings (see Table 1 for more detailed demographic information).

The qualitative analysis identified three major themes for improving the accessibility of neurofeedback: (a) financial support, including insurance coverage; (b) better provider education, including introduction into graduate school curricula; and (c) more research/research funding. Within these themes, participants spoke broadly and theoretically about what would enhance accessibility. They also provided specific strategies they believed would effective. Table 2 includes some example participant quotes within each of the three themes.

Participants said that financial support for clients, providers, or both clients and providers would increase accessibility of neurofeedback treatment. Participants spoke extensively about the cost of neurofeedback to both providers and clients. Cost to providers included the expense of purchasing the equipment and provider training and consultation. The primary expense to clients was the high cost of services often not covered by insurance. One participant specifically mentioned that they were unlikely to have begun implementing neurofeedback were it not for financial support they received through a scholarship program. Some participants mentioned specific ideas, such as the use of local or philanthropic grants, to make neurofeedback accessibility to disadvantaged populations. Most participants said that consistent coverage/reimbursement from health insurance companies would significantly improve neurofeedback’s accessibility for patients. However, some participants said that reimbursement rates would need to be competitive for insurance coverage to have an impact.

### Education

Participants mentioned the need for better neurofeedback provider education. Some reported believing that the way provider education works currently creates barriers to the recruitment and retention of new neurofeedback providers. To date, most neurofeedback training is provided by a variety of software and equipment companies. Most of these trainings focus on the specific type of neurofeedback software and equipment sold by these companies with little information regarding other neurofeedback modalities. Some mentioned specific strategies to remedy this issue, such as a centralized location provided by leaders within the neurofeedback field with information regarding getting started and the basics of different types of neurofeedback and equipment available for purchase. The commercialization of neurofeedback and neurofeedback equipment were cited as a barrier that this strategy could help remedy. Most training is provided to market the companies’ products specifically. Several participants mentioned specifically that introducing mental health professionals to neurofeedback as a modality more broadly during graduate school would help increase the accessibility of the intervention. One participant spoke in detail about their experience of being introduced to neurofeedback in graduate school, noting the significant impact early graduate school mentors had on their career and their future thriving neurofeedback practice.

### Research

Finally, participants mentioned the need for more research and/or research funding. Some spoke specifically of the idea that more research would result in more credibility which would impact the status of insurance coverage as well as general awareness of the intervention. One participant went as far as to say that it is an essential responsibility of practitioners, not just researchers, to publish their findings toward this end.

## Discussion

The findings of this study fell into three overarching themes: cost (financial support and insurance coverage), education (better provider education and introduction of neurofeedback into graduate programs), and research (more research/research funding). Anecdotally, the least surprising of these findings is cost. Within the field of neurofeedback, issues around cost to clients and providers are frequently discussed, and much debate centers around appropriate business practices that work for both clients and providers. Issues around the accessibility of neurofeedback have been noted in past research (Luctkar-Flude et al., 2019). Luctkar-Flude et al.’s (2019) exploration of neurofeedback for cancer survivors included accessibility as one of the major themes in their findings, and their participants described cost and lack of knowledge among healthcare professionals as barriers to accessing neurofeedback. In an exploration of advantages and disadvantages of neurofeedback perceived by neurofeedback providers, Larson et al. (2010) found that 15 of the 16 identified disadvantages were related to financial aspects of implementation, such as equipment costs, education costs, substantial start-up costs, lack of insurance coverage, and ongoing expenses (Larson et al., 2010).

While new to the neurofeedback literature, our participants’ ideas around introducing neurofeedback to graduate students is not novel within the mental health field more broadly. It has been cited in previous literature that it is common in the mental health field to have efficacious interventions that are largely unknown by clinicians (Kettlewell, 2004). One attempt to remedy this issue occurred in the early 2000’s, when the American Psychology Association created the Task Force on Evidence-Based Interventions (EBI) in School Psychology (generally referred to as the Task Force) to close the gap between research and clinician education (Shernoff et al., 2003).

One study surveyed 97 school psychology programs and concluded “it seems clear that training programs would benefit from more information on EBIs identified by the Task Force as information becomes available” (Shernoff et al., 2003, p. 481). Another study found that 71% of licensed school psychologists surveyed reported a perceived inadequacy of graduate program training in evidence-based interventions (Hicks et al., 2014). The current curriculum standards for the Council for Accreditation of Counseling and Related Education Programs (CACREP) include an entire section of standards centering around “Research and Program Evaluation” (Council for Accreditation of Counseling and Related Education Programs, 2024). Though none have been implemented to date, the standards and structures are in place to implement the introduction of neurofeedback into graduate mental health training programs as prescribed by participants. Future research should explore specific ways to successfully introduce neurofeedback to mental health practitioners in training.

Participants’ report of the need for more research and research funding was not surprising as this has been mentioned in the literature by a variety of neurofeedback practitioners (Fisher et al., 2016; Micoulaud-Franchi et al., 2021; Riesco-Matías et al., 2021; Trocki, 2006; Van Der Kolk et al., 2016). Over the past decade, researchers in the PTSD/trauma field have provided a good model for the trajectory of neurofeedback academic research (Askovic et al., 2023; Choi et al., 2023; Panisch & Hai, 2020; Steingrimsson et al., 2020). Future research will need to include studies exploring neurofeedback with other specifics populations and neurofeedback protocols across the implementation spectrum (BLINDED FOR REVIEW).

## Conclusion

This study’s exploration of neurofeedback providers’ perspectives on how to increase the accessibility of neurofeedback led to rich insights about what elements of accessibility stood out to neurofeedback providers and possible tangible solutions to address the neurofeedback field’s issues of accessibility. The three themes to improve accessibility centered around cost (financial support, insurance coverage), education (better provider education, introduction to neurofeedback in graduate schools), and research (need for more research/research funding). The field of neurofeedback would significantly benefit from both investment in tangible solutions to address inaccessibility of neurofeedback and further exploration into the barriers created by the issues of accessibility explored in this study.

## Figures and Tables

**Table 1:** Interview participant characteristics (n = 17)

Characteristics	N (%)
**Race/Ethnicity**	
White	15 (88.2)
Mixed race & Other	2 (11.8)
**Age**	*avg 52.9*
30–39	5 (29.4)
40–49	1 (5.9)
50–59	4 (23.5)
60+	7 (41.2)
**Gender identity**	
Female	13 (76.5)
Male	4 (23.5)
**Years of practice**	*avg 17.8*
0–5	2 (11.8)
6–9	2 (11.8)
10–19	7 (41.2)
20–29	3 (17.6)
30–39	3 (17.6)
**Type of practice setting**	
Community mental health clinic/agency	2 (11.8)
Solo private practice	11 (64.7)
Group private practice	2 (11.8)
Other outpatient setting	2 (11.8)
**Years practicing neurofeedback**	*avg 8.0*
0–5	8 (47.1)
6–9	4 (23.5)
10–19	4 (23.5)
20+	1 (5.9)

**Table 2:** Themes & Example Quotes

Theme	Example Participant Quote
Financial support	*But some type of financial assistance depending upon what the population is that you’re serving…that’s very different than somebody who’s working at a homeless shelter, trying to help people that are chemically dependent, or, you know, or me working in a rural public school district that doesn’t have a lot of funding. I think that if there was a way to get assistance, like specific grants, or I don’t know, you know, just I haven’t really thought about it too much, but I think that would put that would make it, we would definitely see it in more school districts if that could happen*.
	*I don’t like that neurofeedback is only accessible to a certain type of person. And usually that has to do with the financial resources or the time or the… In other words, it’s a privilege, right? You need to have a certain amount of privilege to have neurofeedback accessible. I find the… I just find getting certified in neuro feedback is too rigorous and too it’s too inaccessible unless you are maybe later on in your career*.
Insurance coverage	*Insuranc*e*, get it covered by insurance, and then you’ll have more doctors hear about it, and you’ll have more clients who can do it financially. So, I think that’s the big one*.
	*I think buy in from insurance companies, buy in from Medicaid and Medicare. You know, it’s hard to say for equipment to be cheaper because I know that it costs so much to develop it*.
Better provider education	*If we had kind of a central location to compare systems and what they can do…letting people know what system is good for what. Having more people willing to share openly about their experiences with their equipment and their experiences with neuro feedback in general*.
	*Yeah, um, so right now, it’s tricky. As a practitioner, who is maybe neurofeedback curious, alright, how would you go about adding this to your practice?… And so I feel like there needs to be more resources on, for clinicians, on how like, Okay, now you’re neurofeedback curious, what do you do? What are your next steps? And so I’m a BCIA [Biofeedback Certification International Alliancejmentor, and I’ve had a couple of people approached me for mentoring. And that’s always where we start is just, what’s your goal here? Like, what do you want to do? Do you have equipment and what people do you want to see, to use this equipment with? So I just wish that there were more resources available*.
Introduction of neurofeedback into graduate school curriculum	*I think if enough people get trained, eventually this will become mainstream, just like anything else. But I think it has to start in schools. I think when we have psychology programs, it needs to be there, and* I *think if students can experience that and understand it there will be a bigger drive to each person individually to figure out how to make it work for them*.
	*From the certification organizations like the BCIA or the ISNR [International Society of Neuroregulation and Research], I don’t know how much outreach they do into academic programs. But that seems like it would be a good place too. Like, every college that offers a Master’s in Counseling or Clinical Social Work should probably have a day or a class or something that discusses, you know, neurofeedback or the sort of biological side of treating mental health. And if more colleges were to offer certification programs*.
More research/research funding	*Maybe that has to systemically start at a higher level with more research. So, more research, which then trickles down to academics, which can then present it to the students who can have an interest in it*.
	*I would also just mention the state of the research on it, and that in order for it to be taken seriously more, of course, we do need more studies. And we need that to be a focus of the field, a collaborative focus of the field somehow*.
